# Actual and potential cost effectiveness of off-pump coronary artery bypass grafting in a high-volume cardiothoracic centre

**DOI:** 10.1186/1749-8090-8-S1-O194

**Published:** 2013-09-11

**Authors:** A Page, U Benedetto, E Wlodeck, M Codispoti

**Affiliations:** 1 Papworth Hospital, Cambridge, UK

## Background

OPCAB has been shown to be clinically safe and effective, when compared to conventional CABG. However, there is very little data on the cost-effectiveness of OPCAB in unselected patients undergoing myocardial revascularisation in the current era. Therefore, we sought to investigate the impact of OPCAB on perioperative costs in a large cohort of patients undergoing CABG in a high-volume cardiothoracic centre.

## Methods

We analysed the results of 3595 on-pump versus 348 consecutive OPCAB procedures performed by a single surgeon in a high-volume adult cardiothoracic centre by propensity score matching. Hospital costs related to the perioperative care of patients in the two groups were compared, including cost of disposables, blood products, length of stay in theatre, intensive care unit (ICU) and ward areas. Crude and adjusted differences are reported.

## Results

Clinical outcomes were comparable between the two groups. However, OPCAB was associated with a reduced cost per patient in terms of theatre use (crude -£ 478 / adjusted -£ 225), ICU (-£ 443 / -£ 295), ward stay (-£ 66 /- £ 17) and blood products requirement (-£ 112 / -£ 102 [95% CI -152 to -53 ]). Overall, the adjusted cost saving per patients was -£ 1337 (95% CI -1938 to -737).

## Conclusion

OPCAB is associated with a significant reduction of hospital costs, when compared to on-pump CABG. If OPCAB was adopted by all surgeons performing CABG in a high-volume centre (approximately 1000 CABG procedures/year), the cost effectiveness of OPCAB could result in savings of £ 1.5 million/year.

